# Risk Factors of Mortality in Hospitalized Children with Severe Acute Malnutrition

**DOI:** 10.1007/s12098-019-03016-0

**Published:** 2019-07-06

**Authors:** Dhilip Kumar, Sunil Kumar Rao, Ashok Kumar, Tej Bali Singh

**Affiliations:** 1 Department of Pediatrics, Institute of Medical Sciences, Banaras Hindu University, Varanasi, Uttar Pradesh India; 2 Division of Biostatistics, Department of Community Medicine, Institute of Medical Sciences, Banaras Hindu University, Varanasi, Uttar Pradesh India

*To the Editor:* Childhood undernutrition continues to be a significant health problem in resource-limited settings, contributing to morbidity, mortality and a frequent cause of hospitalization [1]. Children with severe acute malnutrition (SAM) die early, generally within 48 h of hospital admission [2, 3], before the impact of nutritional rehabilitation is visible in them [4]. Better understanding of risk factors of mortality will be helpful in reducing the mortality by timely recognition and specific intervention in these children. There is paucity of studies on predictors of early death in hospitalized SAM children in India.

We conducted an observational study from September 2016 through May 2018 to identify risk factors of mortality in hospitalized children, aged 6 to 60 mo, fulfilling the WHO criteria of SAM. Children with preterm birth or intrauterine growth retardation (IUGR) at birth, inborn error of metabolism, congenital anomalies, chronic renal failure, cerebral palsy, chronic liver disease, and chromosomal abnormalities were excluded. One hundred and twenty two children with SAM were assessed for eligibility. One hundred and ten children met the inclusion criteria and 12 were excluded. Of these 110 children, 57 (51.8%) had edematous malnutrition and 53 (48.1%) had non edematous malnutrition. Out of total children, 90 (81.8%) were discharged from the hospital, 18 (16.6%) died and 2 (1.8%) left against medical advice (LAMA, excluded from the analysis). Males constituted 72 (65.5%) of the children; 51 (46.6%) of the children were in age group of 13 to 36 mo. Nine (50%) children died within 3 d and 16 (88.9%) within 5 d of hospitalization. More children died in edematous group than in non edematous group [12 (21%) *vs*. 6 (11.3%); OR, 2.18]. More than half (*n* = 10) of the deaths occurred in age group 13–16 mo. Risk factors of mortality were analyzed on binary logistic regression for independent association, and it was found that children with fatal outcome were 11.29 times more likely to have shock (*p* = 0.001), 10.2 times more likely to have dehydration (*p* < 0.001), 17.2 times more likely to have acute kidney injury (*p* < 0.001) and 7.1 times more likely to have hyponatremia (*p* = 0.01). The presence of acute diarrhea had six times the odds of mortality (*p* < 0.001).

In this study we found that features of shock, severe dehydration, oliguria and hyponatremia were independent predictors of mortality. The presence of one or more of these risk factors in children with SAM should alert the physician of increased mortality risk in these children and requires early stabilization, close monitoring, and appropriate therapy.

## References

[1] Bhutta ZA, Berkley JA, Bandsma RHJ, Kerac M, Trehan I, Briend A (2017). Severe childhood malnutrition. Nat Rev Dis Primers.

[2] Ahmed T, Ali M, Ullah MM (1999). Mortality in severely malnourished children with diarrhea and use of a standardized management protocol. Lancet.

[3] Maitland K, Berkley JA, Shebbe M, Peshu N, English M, Newton CRJC (2006). Children with severe malnutrition: can those at highest risk of death be identified with the WHO protocol. PLoS Med.

[4] Heikens GT (2007). How can we improve the care of severely malnourished children in Africa. PLoS Med.

